# Myristic Acid Inhibits the Activity of the Bacterial ABC Transporter BmrA

**DOI:** 10.3390/ijms222413565

**Published:** 2021-12-17

**Authors:** Kristin Oepen, Hüseyin Özbek, Anja Schüffler, Johannes C. Liermann, Eckhard Thines, Dirk Schneider

**Affiliations:** 1 Department of Chemistry, Johannes Gutenberg-University, 55128 Mainz, Germany; khackme@uni-mainz.de (K.O.); liermann@uni-mainz.de (J.C.L.); 2 Institut für Biotechnologie und Wirkstoff-Forschung gGmbH (IBWF), 55128 Mainz, Germany; Hozbek@tutanota.com (H.Ö.); schueffler@ibwf.de (A.S.); thines@uni-mainz.de (E.T.); 3 Institute of Molecular Physiology, Johannes Gutenberg-University, 55128 Mainz, Germany

**Keywords:** ABC transporter, BmrA, membrane transport, myristic acid, inhibitor

## Abstract

ATP-binding cassette (ABC) transporters are conserved in all kingdoms of life, where they transport substrates against a concentration gradient across membranes. Some ABC transporters are known to cause multidrug resistances in humans and are able to transport chemotherapeutics across cellular membranes. Similarly, BmrA, the ABC transporter of *Bacillus subtilis,* is involved in excretion of certain antibiotics out of bacterial cells. Screening of extract libraries isolated from fungi revealed that the C14 fatty acid myristic acid has an inhibitory effect on the BmrA ATPase as well as the transport activity. Thus, a natural membrane constituent inhibits the BmrA activity, a finding with physiological consequences as to the activity and regulation of ABC transporter activities in biological membranes.

## 1. Introduction

Membrane integral transport proteins mediate and control the translocation of essential compounds across biological membranes, involving the uptake and efflux of sugars, inorganic ions, nucleotides or drugs [1]. Primary active transporters use the energy gained via ATP hydrolysis for transport [1,2], and such transport ATPases are typically represented by ion pumps and ATP-binding cassette (ABC) transporters [2]. ABC transporters can be found in all kingdoms of life and they either import or export substrates against a concentration gradient. While, in bacteria, ABC importers and exporters can be found, eukaryotes mainly contain exporters [1,3]. Structurally, all ABC transporters consist of two nucleotide binding domains (NBDs) and two transmembrane domains (TMDs). The TMDs consist of α-helix bundles that mediate the actual TM flux of the substrates, whereas ATP is hydrolyzed in the NBDs [3,4,5]. In contrast to the NBD, the sequences of the TMDs are typically less conserved and the TM topology can vary. The four domains of an ABC transporter can be part of a single polypeptide chain, or the transporter assembles from two to four individual subunits [5,6]. One TMD and one NBD can be fused to form a so-called half-transporter, which can either form a homodimeric (identical half-transporters) or assemble as a heterodimeric (different half-transporters) full-transporter [1,3,7,8].

Many ABC transporters appear to be involved in bacterial multidrug resistances [9]. For example, in cervimycin C resistant *Bacillus subtilis* colonies the *bmrA* gene, encoding an ABC half-transporter, was strongly overexpressed, which led to the assumption that BmrA (*Bacillus* multidrug-resistance ATP) is able to effectively transport this antibiotic out of the cell [10,11]. Besides cervimycin C, BmrA can transport a broad range of substrates, which include Hoechst 33342, doxorubicin as well as 7-aminoactinomycin D [11].

BmrA, a homodimeric ABC transporter with a molecular mass of 64.9 kDa, is homologous to the bacterial ABC transporters LmrA and MsbA [6,12] as well as to the human P-glycoprotein [3,11]. In recent years, BmrA became a paradigm for studying ABC transporters, mostly due to the vast number of seemingly unrelated substrates as well as its homology to the human P-glycoprotein [11,12,13].

As BmrA is involved in excretion of certain antibiotics out of bacterial cells, we aimed at identifying small molecules which inhibit the BmrA transport activity and thus might be used to modulate the BmrA transport activity. Screening of extract libraries isolated from fungi revealed that the C14 fatty acid myristic acid has an inhibitory effect on the BmrA ATPase as well as the transport activity. Thus, BmrA “sleeps with the enemy”, as a natural membrane constituent inhibits its activity, a finding with physiological consequences as to the activity and regulation of ABC transporter activities in biological membranes.

## 2. Results

### 2.1. Myristic Acid Inhibits the ATPase Activity of the ABC Transporter BmrA

**Figure 1:**
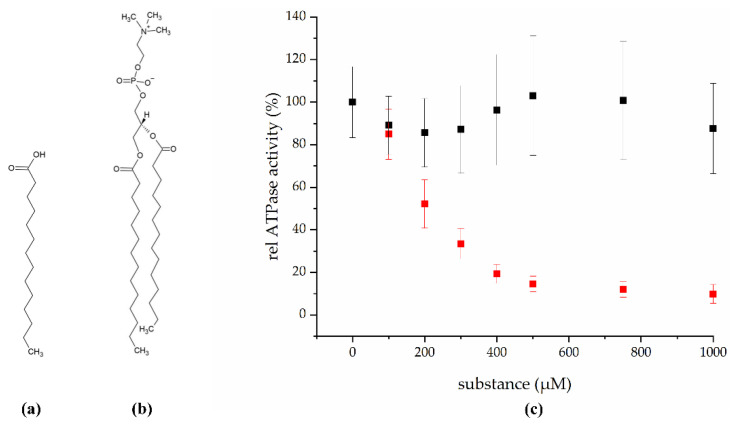
Inhibition of the BmrA ATPase activity by myristic acid. (**a**) Structure of the fatty acid myristic acid (C_14_H_28_O_2_). (**b**) Structure of 1,2-dimyristoyl-sn-glycero-3-phosphocholine (DMPC), a membrane phospholipid containing two myristic acids as fatty acids. The structures were created using ChemSketch [14]. (**c**): Myristic acid (*n* ≥ 6, ± SEM; red) inhibits the in vitro ATPase activity of detergent solubilized BmrA, whereas the lipid 1,2-dimyristoyl-sn-glycero-3-phosphocholin (DMPC, *n* = 12, ± SEM; black) does not.

In total, 448 fungal extracts were tested for an inhibitory effect on the BmrA ATPase activity and 22 were found to be active. One of the most promising candidates, the mycelial extract of strain IBWF 030-11, was selected for further characterization. To identify the natural product responsible for the inhibitory effect, the fungus was cultivated in a 20 L scale and the active principle was isolated. Based on NMR analysis, the isolated substance was identified as the C14 fatty acid myristic acid (Figure 1a), 0.25 mg of which was isolated per L axenic fungus culture.

To quantitatively evaluate the inhibitory potential of myristic acid, we next examined the in vitro ATPase activity of BmrA at increasing myristic acid concentrations (Figure 1c, red). The BmrA ATPase activity was determined in buffer containing myristic acid concentrations ranging from 0 to 1000 µM to calculate the IC_50_ value, i.e., the substance concentration required to inhibit 50% of the protein’s ATPase activity. The ATPase activity of BmrA in pure 5 mM DDM was 1.0 ± 0.16 µmol/min per mg protein, a value comparable to values previously determined under slightly different experimental conditions [15,16]. As expected in the presence of an inhibitor, the ATPase activity constantly decreased with increasing myristic acid concentrations, until at ~500 µM myristic acid the activity levelled off to about 12%. Based on this analysis, the turning point, i.e., the IC_50_, is at approximately 200 µM myristic acid. Furthermore, these results additionally indicate that myristic acid is not a BmrA substrate that stimulates the ATPase activity, as has been observed with other ABC transporter substrates [17,18,19].

Nevertheless, the concentration for free myristic acid within the membrane is low *in vivo*, as myristic acid typically is part of diacylglycerol lipids. This now raised the question whether the ATPase activity of BmrA is also affected by myristic acid-containing phospholipids. Thus, we next tested the in vitro ATPase activity of isolated BmrA in the presence of increasing concentrations of 1,2-dimyristoyl-sn-glycero-3-phosphocholine (DMPC, Figure 1b,c black). DMPC is a glycerophospholipid, containing two myristic acids as fatty acids attached to the glycerol backbone. As the determined ATPase activity of BmrA is not significantly affected by DMPC, the inhibitory effects observed before can clearly be linked to the isolated myristic acid.

### 2.2. The Stability of BmrA in Micelles Is Not Affected by Myristic Acid

Myristic acid has detergent properties and can form micelles in solution [20]. As harsh detergents can unfold (membrane) proteins [21], at least to some extent, the question arose whether myristic acid does not inhibit the BmrA ATPase activity via binding but via denaturation of the protein structure, resulting in a diminished protein activity.

As changes in a local tryptophan environment, e.g., caused by protein denaturation, result in a different fluorescence emission spectrum, the stability of purified BmrA in DDM micelles can be determined by fluorescence spectroscopy. A well-established approach to unfold membrane proteins is to solubilize the protein in a mild detergent, such as DDM, and to subsequently titrate in increasing amounts of a harsh detergent, typically SDS [22,23]. Addition of SDS results in formation of mixed DDM/SDS micelles, which eventually unfold α-helical membrane proteins. It is noteworthy that, while the mixed micelles can indeed unfold soluble regions or domains of membrane proteins, the membrane integral protein parts typically retain their helical structure, and the term “unfolding” here in fact describes the separation of previously interacting individual TM α-helices [24].

**Figure 2:**
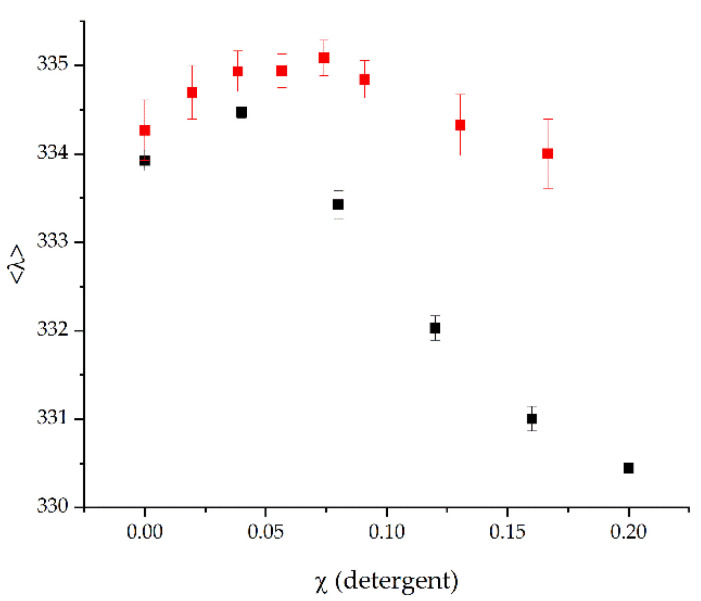
Myristic acid does not destabilize the BmrA structure as SDS. Increasing amounts of myristic acid (*n* = 9, ± SEM; red) or SDS (*n* = 7, ± SEM black) were titrated to BmrA solubilized in DDM micelles. While addition of SDS leads to a larger decrease in the average emission wavelength (<λ>), this was not observed when myristic acid was added.

When the purified protein was exposed to increasing SDS concentrations, the average emission wavelength (<λ>) decreased (Figure 2, black). At a low SDS mole fraction of χ_SDS_ = 0.04 the <λ> slightly increased, a behavior also observed with other TM proteins [25], whereas higher SDS concentrations led to a dramatic decrease in the average emission wavelength. In contrast, while addition of low myristic acid concentration also resulted in a slight increase in the <λ>, further increasing the myristic acid concentration resulted in a slightly decrease, albeit the <λ> myristic acid never changed to an extent as observed with SDS. This indicates that myristic acid does not substantially destabilize the protein, in contrast to SDS.

### 2.3. Myristic Acid Inhibits the BmrA-Mediated Transport of Hoechst 33342 in Inverted Membrane Vesicles

Hoechst 33342 is a substrate commonly used when the BmrA activity is studied in inverted membrane vesicles. Upon spontaneous membrane partitioning, the dye’s fluorescence increases. When the dye is actively transported out of the lipid membrane by BmrA and expelled to the liquid surrounding, the fluorescence decreases again. Importantly, the transport of Hoechst 33342 depends on the BmrA ATPase activity [11].

**Figure 3:**
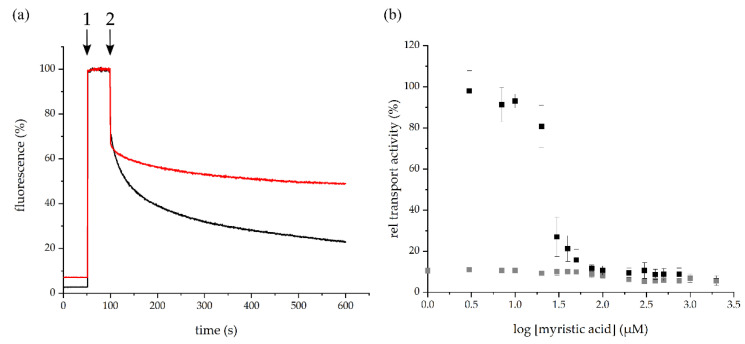
Myristic acid inhibits Hoechst 33342 transport in inverted C41 (DE3) *E. coli* membrane vesicles. (**a**) Kinetics of Hoechst 33342 transport followed in absence (black) or presence of 1000 µM myristic acid (red), 1: addition of Hoechst 33342, 2: addition of ATP. (**b**) 3–1000 µM myristic acid was added to inverted membrane vesicles prepared from BmrA expressing cells (black) or *E. coli* C41(DE3) cells transformed with an empty vector (grey) and Hoechst 33342 transport was quantified (*n* = 3, ± SEM).

At first, the initial fluorescence of the inverted membrane vesicles was monitored in absence of Hoechst 33342 (Figure 3a). Subsequently, upon addition (Figure 3a, 1) and membrane incorporation of Hoechst 33342, the fluorescence increased tremendously due to membrane partitioning of the dye [26]. After addition of ATP the fluorescence intensity decreased again due to the removal of Hoechst 33342 from the membrane. The decrease in the fluorescence intensity thus directly correlates with the BmrA transport activity [26]. When the BmrA-mediated Hoechst 33342 transport was measured in inverted membrane vesicles pre-incubated with myristic acid, the initial fluorescence intensities were similar. Upon ATP addition (Figure 3a, 2), the fluorescence intensity first decreased, as observed in absence of myristic acid, yet remained on a higher fluorescence level. This implies that more Hoechst 33342 molecules remained incorporated within the lipid bilayer, and thus, less Hoechst 33342 molecules were transported by the ABC transporter (Figure 3a, red). For comparison, the absolute value of the slope was used to quantify the transport activity. The BmrA wild type transport activity was set as 100% and the generated values at increasing myristic acid concentrations were normalized to the wild type (Figure 3b).

To test a (potential) inhibition of the BmrA transport activity, myristic acid was added to inverted membrane vesicles at increasing concentrations (3–1000 µM) and the Hoechst 33342 transport was quantified (Figure 3b). The BmrA transport activity was essentially not affected up to myristic acid concentrations of 10 µM. However, at 20–50 µM myristic acid, the transport activity of BmrA massively decreased, and at around 100 µM myristic acid a plateau was reached. Based on a Boltzmann fit, an IC_50_ value of about 25 µM myristic acid was determined for the Hoechst transport using inverted vesicles. As expected, the inverted vesicles prepared from *E. coli* C41(DE3) cells transformed with an empty vector did not show activity at any given myristic acid concentration.

**Figure 4:**
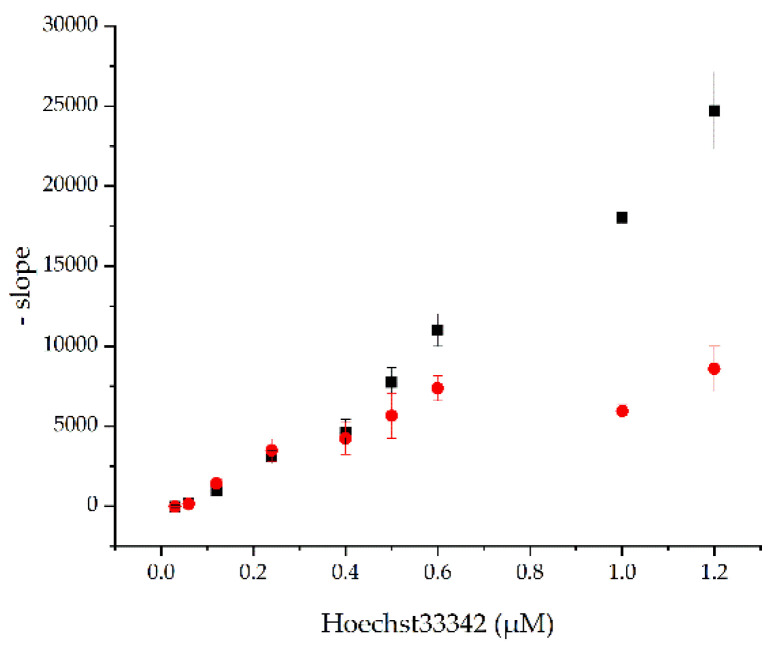
BmrA-mediated Hoechst 33342 transport at a constant myristic acid concentration. 0.2 mM myristic acid (red) or methanol (control; black) were added to inverted membrane vesicles and Hoechst 33342 transport was tested at increasing Hoechst 33342 concentrations (*n* = 3, ± SEM).

Yet, myristic acid might not inhibit the BmrA transport activity, but in fact is a substrate that simply competes with Hoechst 33342 for transport. To test this assumption, inverted vesicles were first exposed to 0.2 mM myristic acid, and the Hoechst 33342 transport was measured as before, but at different Hoechst 33342 concentrations. Upon addition of ATP, the absolute value of the slope of the fluorescence decrease was determined at each Hoechst 33342 concentration (Figure 4). When Hoechst 33342 and myristic acid compete for transport, addition of small amounts of Hoechst 33342 should not result in a measurable transport activity, and only at rather high Hoechst 33342 concentrations is an activity expected to be observed. As can be seen in Figure 4, the absolute value of the slope increased linearly with increasing Hoechst 33342 concentrations for the control (no myristic acid). In contrast, the activity of the inverted vesicles containing a constant myristic acid concentration but increasing Hoechst 33342 concentrations increased linearly up to a concentration of ~0.6 µM Hoechst 33342. At higher Hoechst 33342 concentrations, the initial slope remained more or less constant. If Hoechst 33342 and myristic acid were both substrates competing for binding and translocation, an initial significantly slowed down Hoechst 33342 transport would be expected at the (high) constant myristic acid concentration. However, this was not observed. Instead, the transport activity leveled off at a rather low Hoechst concentration. Consequently, the data indicate that there is no simple competitive or non- or uncompetitive inhibition of BmrA by myristic acid. The BmrA activity is inhibited by myristic acid via a rather complex mechanism. Noteworthy, the fluorescence intensities (without ATP added) of the control and the myristic acid-incubated inverted vesicles were more or less equal for each Hoechst 33342 concentration. The increasing amounts of Hoechst 33342 lead to a linearly increasing slope (data not shown).

### 2.4. Myristic Acid Does Not Solubilize Overexpressed BmrA in Inverted Vesicles

**Figure 5:**
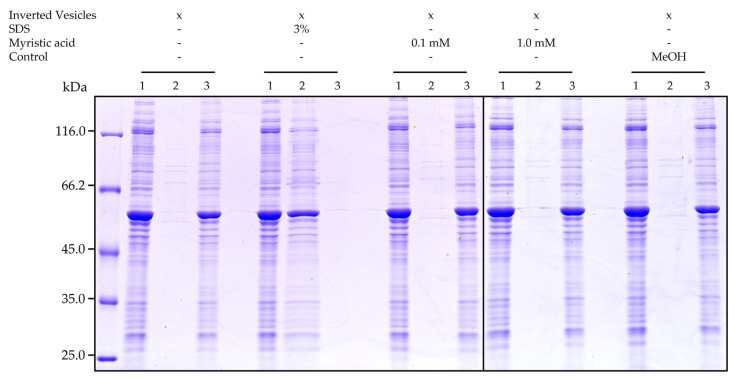
Stability of inverted membrane vesicles with overexpressed BmrA. Inverted vesicles were exposed to either SDS (3% (*w*/*v*), myristic acid (0.1 mM or 1.0 mM dissolved in methanol) or methanol (0.5% (*v*/*v*). Intact membranes were found in the pellet, whereas solubilized membrane proteins were found in the supernatant (solely when SDS was added). 1: BmrA and membrane proteins in inverted vesicles not influenced by any substances. 2: Supernatant after ultracentrifugation containing solubilized BmrA (and other membrane proteins). 3: Pellet after ultracentrifugation comprising of inverted vesicles with overexpressed BmrA. This experiment was performed three times with three different inverted membrane vesicle preparations, which all showed the same results.

Due to the detergent properties of myristic acid, it was possible that the fatty acid solubilized the overexpressed protein in the inverted vesicles, resulting in the observed decreased BmrA transport activity. To finally exclude this, vesicles were incubated with the detergent SDS or myristic acid for 1 h. Subsequently, solubilized protein was separated from membranes via ultracentrifugation, and solubilized proteins were analyzed via SDS PAGE. As can be seen in Figure 5, while BmrA was properly solubilized by SDS, neither at low nor at high myristic acid concentrations BmrA was extracted from the membranes. Thus, the inverted vesicles remained intact at the here analyzed myristic acid concentrations.

## 3. Discussion

In the present study, we identified myristic acid as a potential inhibitor of the BmrA ATPase and transport activity. This saturated C14 fatty acid (Figure 1a) is widely distributed in plant and animal fat and can naturally be found in high concentrations in coconut oil as well as in butter fat. Furthermore, myristic acid is utilized in the food industry as multifunctional food additive and flavor excipient [27]. In *Bacillus subtilis,* around 3.6% of all lipids are myristic acid [28]. Yet, myristic acid inhibits the BmrA activity exclusively as a free acid, but not when part of phospholipids (Figure 1c, black). Thus, while 3–4% of all lipids in *Bacillus subtilis* are myristic acid, most of these will be part of di- or even triacylglycerols and thus the concentration of the inhibiting species, i.e., the free acid, will be low.

As we have shown here, myristic acid does neither inhibit the BmrA activity indirectly, via destabilizing membranes and extracting the protein from membranes (Figure 5), nor via detergent-induced denaturation of the protein (Figure 2). Thus, myristic acid appears to directly inhibit the BmrA ATPase and transport activities (Figure 1c and Figure 3b), albeit the inhibitory mechanism appears to be complex.

It is well known that, e.g., the detergent Triton X-100 stimulates the ATPase activity of the ABC transporter P-glycoprotein [29], and based on this and other observations, it has been concluded that detergents can serve as P-glycoprotein substrates [30]. In many cases, addition of substrate even increases the ATPase activity of ABC transporters, which was, however, not observed here. ABC transporter substrates have rather diverse structures [11], and also fatty acids are transported by ABC transporters [31]. This has been shown for some ABC transporters, such as MsbA or LmrA [32,33]. When the lipid A ABC transporter MsbA was heterologously expressed in *L. lactis* cells, it has been observed that myristic acid might be transported due to no change in the determined IC_50_ value [33]. Yet, based on the results presented here (Figure 4), myristic acid appears not to simply compete with Hoechst 33342 for the substrate binding site and transport.

But why is the determined IC_50_ value so much higher when the ATPase activity was monitored than when the transport activity was monitored? Although we cannot ultimately answer this question, these two measurements can only be compared to some extent. While we have a well-defined protein and detergent concentration when the isolated protein was analyzed, this was not the case in the inverted vesicles. Furthermore, in inverted vesicles we might have other components that interact with myristic acid. Yet, this would probably reduce, and not increase, the inhibitory activity of myristic acid. In bacterial membranes, most membrane lipids are not part of bulk lipids, but are (more or less tightly) bound to membrane proteins (reviewed [34]). Thus, the concentration of myristic acid added to the lipid phase is probably much higher at any given total myristic acid concentration, compared to the situation in micelles. In the latter, the myristic acid will incorporate into free micelles as well as into BmrA-containing micelles to form mixed micelles. Thus, myristic acid is likely highly diluted in the micellar system, which results in a rather high IC_50_ value for the determined ATPase activity. Finally, it is also possible that myristic acid induces a conformational change or decoupling of the NBD and TMD in the lipid environment, as e.g., observed with the mutant BmrA E474R [35].

## 4. Materials and Methods

### 4.1. Screening for BmrA Inhibitors using Fungal Extracts and Isolation of Myristic Acid from IBWF 030-11

448 fungal extracts dissolved in DMSO were tested in 384 well plate format in accordance to assay methodology described in 4.6 at a concentration of 0.5 mg/mL in initial screens. NADH decrease was monitored with a multilabel reader (Perkin Elmer Envision 2104). Among the active extracts was one isolated from the mycelium of strain IBWF 030-11 (*Clavicipitaceae*). This strain is deposited in the strain collection at the *Institut für Biotechnologie und Wirkstoff-Forschung* (IBWF), Mainz. The active principle was identified by subfractionation of the initial extract and retesting whereby the activity was tracked to a natural product which was unknown to the IBWF compound library. To isolate the active principle for structure elucidation and natural product characterization, strain IBWF 030-11 was regrown and cultivated in a 20 L fermenter in YMG medium (YMG: 4 g yeast extract, 10 g malt extract, 10 g glucose, pH was adjusted to 5.5 before autoclaving). For inoculation, a well-grown flask culture in the same medium was used. The mycelium was separated from the culture by filtration 16 days after inoculation, freeze-dried (dry weight 122 g) and subsequently extracted with MeOH to yield 24 g crude extract. Flash chromatography on silica gel 60 (0.04–0.063 mm; Macherey-Nagel) yielded a subfraction (750 mg) which was applied to a second silica gel fractionation to yield intermediate 1 (628 mg). Preparative HPLC (PrepHT Zorbax Eclipse XDB-Phenyl, 5 µm, 21 × 250 mm, Agilent Technologies, MeCN: 0.1% TFA in H_2_O gradient from 50% MeCN to 70%MeCN in 20 min, 21 mL/min) of intermediate 1 yielded 5 mg of the active substance (myristic acid (RT 11.5 min)). The purity of the active substance was checked with mass spectrometry.

### 4.2. NMR Analysis

Myristic acid was identified by ^1^H, ^13^C, COSY, HSQC, and HMBC NMR using a Bruker Avance III 600 MHz spectrometer, equipped with an inverse Helium-cooled cryoprobe. All shifts are given relative to TMS, using the residual CHCl_3_ shift as reference (7.26 ppm) [36].

^1^H NMR (600 MHz, CDCl_3_) *δ* = 2.36 (t, *J* = 7.5 Hz, 2H, H-2), 1.63 (pseudo quin, *J* = 7.5 Hz, 2H, H-3), 1.44–1.19 (m, 20H, H-4 to H-13), 0.88 (dist. t, *J* = 6.9 Hz, 3H, H-14) ppm. ^13^C NMR (151 MHz, CDCl_3_) *δ* = 176.6* (C-1, by HMBC), 33.4 (C-2), 31.9 (C-12), 29.8–29.0 (C-4 to C-11), 24.7 (C-3), 22.7 (C-13), 14.2 (C-14) ppm. Shifts are in accordance with the literature [37].

### 4.3. Cloning

The *Bacillus subtilis bmrA* gene was amplified via PCR from genomic *B. subtilis* (strain 168) DNA using the following primers:

Forward: 5′ GCTACCTCTAGAATGCCAACCAAGAAACAAAAATCTAAAAG 3′ and reverse: 5′ GCTATTCTCGAGCCCGGCTTTGTTTTCTAAG 3′.

The PCR product was cloned into the plasmid pET303-CT/His (Invitrogen, Carlsbad, CA, USA), whereby the 3´ end was extended by a sequence coding for a short linker and a His_6_-tag.

### 4.4. Expression

The pET303-CT/His-BmrA plasmid was transformed in competent BL21(DE3) pLysE *E. coli* cells, and cells were subsequently plated on LB agar containing 100 µg/mL ampicillin. A single colony was used to inoculate an overnight culture, which was used the next morning to inoculate a 2 L culture, containing 100 µg/mL ampicillin and 30 µg/mL chloramphenicol. The cells were cultivated at 37 °C with constant agitation (150 rpm). When the culture reached an OD_600_ of ~0.8, protein expression was induced via addition of isopropyl-ꞵ-D-thiogalacto-pyranoside (IPTG) to a final concentration of 0.5 mM. Cells were harvested after 3–4 h via centrifugation (3050× *g*, 10 min at 4 °C). The cell pellets then were stored at −20 °C.

### 4.5. Purification

The cell pellets were resuspended in 50 mM phosphate buffer (pH 8.0), 300 mM NaCl, 10% glycerol (*v*/*v*) and lysed using a microfluidizer (LM20, Microfluidics, Westwood, CA, USA, 18000 PSi). After centrifuging at 12,075× *g* (10 min at 4 °C), the supernatant was centrifuged again at 165,000× *g* for 1 h at 4 °C to isolate membranes. The membranes were solubilized in solubilization buffer (50 mM phosphate buffer (pH 8.0), 300 mM NaCl, 10% glycerol (*v*/*v*) with 1% *n*-dodecyl-ꞵ-D-maltoside (DDM) (*w*/*v*)) to extract the membrane-incorporated proteins. After 1 h of incubation, insolubilized protein was removed by centrifugation (165,000× *g*, 20 min) and the equilibrated Protino^®^ Ni-NTA agarose (2 mL resin/L of *E. coli* culture; Macherey-Nagel GmbH & Co. KG, Düren, Germany) was mixed with the solubilized proteins and incubated for 1 h. After washing the Ni-NTA agarose with 20 mL washing buffer (50 mM phosphate buffer (pH 8.0), 300 mM NaCl, 10% glycerol (*v*/*v*) with 0.1% DDM (*w*/*v*) and 40 mM imidazole), the protein was eluted with 5 mL elution buffer (50 mM phosphate buffer (pH 8.0), 300 mM NaCl, 10% glycerol (*v*/*v*) with 0.1% DDM (*w*/*v*) and 400 mM imidazole). To exchange the buffer to the required assay buffer (containing 5 mM DDM), a PD-10 desalting column (Macherey-Nagel GmbH & Co. KG, Düren, Germany) was utilized. The concentration of the purified membrane protein BmrA was determined photometrically by measuring the absorbance at 280 nm and the calculated extinction coefficient ε = 38850 M^−1^ cm^−1^.

### 4.6. ATPase Activity of Purified BmrA

The ATPase activity of 0.2 µM protein BmrA was measured in DDM micelles at 25 °C in 50 mM Hepes-KOH (pH 8.0 and 5 mM DDM) with 3.5 mM ATP, 10 mM MgCl_2_, 0.28 mM NADH, 2 mM phosphoenolpyruvate, by adding 2 µL of pyruvate kinase (600–1000 U/mL)/lactate dehydrogenase (900–1400 U/mL) mix to the 200 µL test volume. Absorption changes were measured at a wavelength of 340 nm and a slit width of 4 nm using a Lambda 35 U *V*/*V* is spectrophotometer (PerkinElmer, Inc., Waltham, MA, USA). Here the usage of NADH was monitored. The NADH decrease was measured for 180 sec and converted into the BmrA activity in min^−1^.

(1) $$ATPase\;activity=-\frac{\Delta A_{340}}{\Delta t}\cdot \frac{1}{l\cdot \varepsilon_{NADH}}\cdot MW_{BmrA}\cdot \frac{1}{c_{BmrA}}$$

Equation (1) comprises of the slope ($\frac{\Delta A_{340}}{\Delta t}$) of the NADH decrease, the optical pathlength (*l* in cm), the extinction coefficient of NADH (*ε_NADH_* = 6220 M^−1^ cm^−1^) as well as of the calculated molecular weight of BmrA (65584.27 g/mol) and the used BmrA concentration (*c_BmrA_* in g/L). The ATPase activity without substance was set as 100% and the data points measured at different myristic acid and DMPC concentrations were normalized to this level with respect to the error propagation.

For the experiments (except for the screening and NMR), commercially available myristic acid (Sigma-Aldrich, Merck KGaA, Darmstadt, Germany, M3128) with a high purity (≥99%) was used. Myristic acid (dissolved in methanol) or 1,2-dimyristoyl-sn-glycero-3-phosphocholin (DMPC; solved in chloroform) was pipetted at the specified amounts into the reaction tubes. Subsequently, the solvent was removed by a constant stream of nitrogen, and these reaction tubes were further stored for at least 30 min under vacuum. The dried fatty acid was dissolved in sample buffer (50 mM Hepes-KOH, pH 8.0, 5 mM DDM) and the solution was vortexed and further incubated for at least 1 h under constant agitation (500 rpm). Thereafter, the protein was added, and the solution incubated for another 10 min at 25 °C. After adding the other compounds to the solution, the decrease in absorbance was immediately monitored.

### 4.7. Hoechst 33342 Transport Measured Using BmrA-Containing Inverted E. coli Membrane Vesicles

Inverted *E. coli* membrane vesicles were prepared as described in Steinfels et al., 2002 [38], except that a microfluidizer was used at 18000 Psi instead of a French press.

The membrane protein concentration was determined with a BCA assay (Pierce^TM^ BCA protein assay kit, Thermo Fisher Scientific, Rockford, IL, USA), following the vendor´s instructions. In all, 50 µg of inverted membrane vesicles were dissolved in a final volume of 200 µL of 50 mM Hepes-KOH (pH 8.0), 2 mM MgCl_2_, 8.5 mM NaCl, 4 mM phosphoenolpyruvate and 20 µg/mL pyruvate kinase. Myristic acid (dissolved in methanol) was added to the reaction mixture at various concentrations (3 µM, 7 µM, 10 µM, 20 µM, 30 µM, 40 µM, 50 µM, 75 µM, 100 µM, 200 µM, 300 µM, 400 µM, 500 µM, 750 µM, 1000 µM) and following incubation for more than 10 min. The maximal concentration of methanol in this reaction mixture was 0.5% (*v*/*v*). Fluorescence emission was measured at 457 nm using a FluoroMax-4 fluorometer (Horiba Instruments Inc., Edison, NY, USA) upon excitation at 355 nm, with excitation and emission slit widths of 2 nm and 3 nm, respectively. After monitoring the fluorescence for approximately 50 s, the measurement was stopped. Then, 2 µM 2′-[4-ethoxyphenyl]-5-[4-methyl-1-piperazinyl]-2,5′-bis-1H-benzimidazole (Hoechst 33342) was added and the fluorescence was measured again for approximately 50 s. Then, ATP was added to a final concentration of 2 mM and the fluorescence was further monitored for ~500 s. Data points were collected every 0.5 s. The slope of the measured fluorescence intensity (after addition of ATP) was determined by fitting a linear regression line with a large coefficient of determination. Then the absolute value of the slope of the sample containing inverted membrane vesicles with overexpressed BmrA (50 µg) plus methanol (0.5% (*v*/*v*)) was set as “100% transport activity” and the data points measured at different myristic acid concentrations were normalized to this considering error propagation. All presented data are based on three independent inverted vesicle preparations, and the mean with corresponding SEM is shown.

For the competition assay, inverted membrane vesicles containing 50 µg protein were incubated with 0.2 mM myristic acid for at least 10 min at 25 °C. The fluorescence signal was measured as described (exception: slit width of 3 nm). When the measurement was started, the fluorescence was monitored for 50 s. Varying concentrations of Hoechst 33342 were added (0.03 µM, 0.06 µM, 0.12 µM, 0.24 µM, 0.3 µM, 0.4 µM, 0.5 µM, 0.6 µM, 1.0 µM, 1.2 µM) and the measurement and data evaluation continued as described before.

### 4.8. Destabilization of BmrA in DDM Micelles

Purified BmrA (2 µM) in 50 mM phosphate buffer (pH 8.0), 300 mM NaCl, 10% glycerol (*v*/*v*), 5 mM DDM was mixed with increasing amounts of sodium dodecyl sulfate (SDS; concentrations: 0 mM, 0.21 mM, 0.43 mM, 0.68 mM, 0.95 mM, 1.25 mM) or myristic acid (concentrations: 0 mM, 0.1 mM, 0.2 mM, 0.3 mM, 0.4 mM, 0.5 mM, 0.75 mM, 1.0 mM). The samples were incubated at room temperature for 1 h. Fluorescence spectra were recorded (from 290–450 nm) using a FluoroMax-4 fluorometer with 280 nm excitation and a slit width of 3 nm. The average emission wavelength (<λ>), which represents changes in shape and position of the spectrum, was used to characterize the entire measured tryptophan fluorescence emission spectrum. The <λ> was calculated as described in the following Equation (2):

(2) $$\langle \lambda \rangle =\frac{\left(\Sigma_{i}{\;\lambda }_{i}\cdot I_{i}\right)}{\Sigma_{i}I_{i}}$$

Here *λ* is the wavelength in nm and *I* is the fluorescence intensity.

### 4.9. Stability of BmrA in Inverted Vesicles

Inverted vesicles (250 µg/mL) were incubated with either 0.1 mM or 1.0 mM myristic acid for 1 h at room temperature at constant agitation. As a control, 0.5% (*v*/*v*) methanol was added to inverted vesicles. As a second control, vesicles were incubated with 3% SDS (*w*/*v*). After incubation, the samples were centrifuged at 140,000× *g* for 1 h at 25 °C. Samples were taken before incubation with the substances and after centrifugation (from the supernatant as well as the pellet). A total of 10 µL of each sample was analyzed on a 10% SDS PAGE gel.

## 5. Conclusions and Implications

At first, inhibition of an ABC transporter activity by a naturally occurring fatty acid appears to be unexpected, albeit the physiological concentration of myristic acid in *Bacillus subtilis* membranes is probably not sufficiently high to compromise the BmrA transport activity. However, it might even be beneficial to inhibit a basal ATPase activity, by which ATP might be wasted in cells, *in vivo* using naturally occurring membrane incorporated substances, such as myristic acid.

The initial idea of this project was to identify potential modulators of the BmrA activity. Based on the presented results myristic acid might qualify as a drug excipient, as co-application with ABC transporter substrates might reduce the risk of drug export out of a cell. Furthermore, as mentioned in the discussion, myristic acid is used as a multifunctional food additive and flavor excipient [27]. Thus, this food additive has the potential to interfere with (human) ABC transporters, an aspect which, to the best of our knowledge, has never been discussed thus far and which we will explore in future experiments.

## Data Availability

The data presented in this study are available on request from the corresponding author.
